# Supplementary material for: New Perspectives on the Risks of Hydroxylated Polychlorinated Biphenyl (OH-PCB) Exposure: Intestinal Flora α-Glucosidase Inhibition
Source: Toxics. 2024 Mar 24;12(4):237. doi: 10.3390/toxics12040237 (PMC11053903; doi:10.3390/toxics12040237)

Supplementary Information

Supplementary Figure S1 Kinetic parameters (Km, Vmax) of PNPG catalyzed by α-glucosidase.

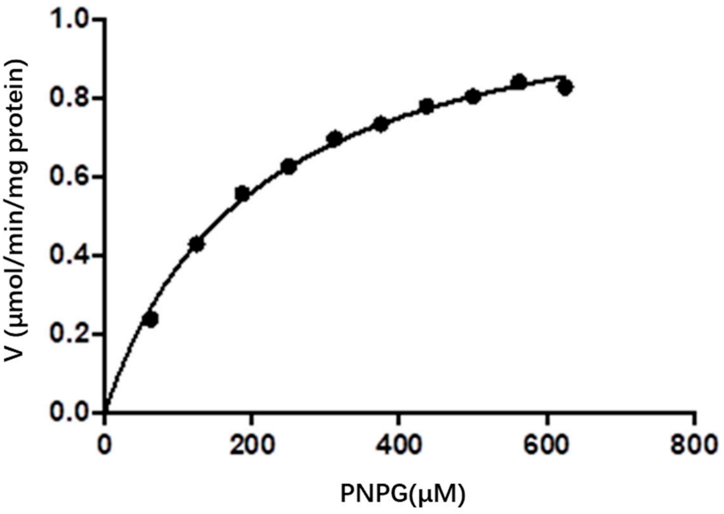

Supplement: Supplementary file 1 [file toxics-12-00237-s001.zip › toxics-2883187-supplementary.pdf]
